# Comparison of Nursing Performance Competencies and Practical Education Needs Based on Clinical Careers of Operating Room Nurses: A Cross-Sectional Study

**DOI:** 10.3390/healthcare8020136

**Published:** 2020-05-18

**Authors:** Ji Hye Jung, Hye Jin Kim, Ji-Su Kim

**Affiliations:** 1 Uijeongbu ST. Mary’s Hospital, Gyeonggi-do 11765, Korea; salang235@naver.com; 2 Department of Nursing, Chung-Ang University, Seoul 06974, Korea; caunurse@naver.com

**Keywords:** nurse, nurses performance evaluation, nursing education, clinical competence, operating room nursing

## Abstract

In order to provide appropriate practical training for quality nursing care, it is necessary to evaluate nursing performance competencies in terms of clinical careers. Using convenience sampling, this cross-sectional study aimed to identify the nursing performance competencies and practical education needs of operating room nurses by evaluating nurses’ clinical careers. The participants in this study were 182 nurses working in the operating rooms of four general hospitals in Seoul, Korea. Data came from self-report questionnaires regarding nursing performance competencies and practical education needs. The results showed that participants’ nursing performance competencies and practical education needs differed significantly across clinical career groups. Further, participants’ nursing performance competencies were negatively correlated with practical education needs in terms of clinical career. In order to develop an effective and continuous practical education program for operating nurses, various education programs that reflect nursing performance competencies and practical education needs for particular stages of clinical careers are necessary.

## 1. Introduction

Operating rooms (ORs) are the primary locations for the application of advanced technologies and are high-risk medical areas with a high concentration of patients [1]. In the OR, an improvement in healthcare providers’ professionalism is directly related to patient safety and health-related outcomes [2]. Fesco et al. [3] reported that superior technical performance positively affects patient outcomes such as intraoperative complications, morbidity, and mortality in systematic review. Specially, OR nurses’ responsibility for patient safety has expanded and now encompasses the periods before and after surgical operations [4].

Nursing competence is a fundamental aspect of safe clinical practice [5]. Similarly, OR nurses’ competence is indispensable to ensure patient safety during the perioperative process [6]. Waston et al. [7] suggested that the assessment of nursing performance competencies was important in identifying areas that require additional professional development and education. To improve nursing, these competencies must be continuously developed through appropriate education programs [8]. In addition, assessment of practical education needs can contribute to achieving learner-oriented education and increasing the range of education provided; it can also identify educational areas that should be prioritized [7]. Brown [9] reported that education needs assessments can be used to create future education plans and assessment criteria as well as suggest areas of education that require investment. Thus, assessing practical education needs could help to improve nursing competency, increase learning efficiency, and examine individual nursing performance competencies; furthermore, assessment has a direct relationship with patient safety [2,10,11].

Recent studies conducted in Korea regarding individual nursing performance competencies have focused on hospitals as a whole, conjoining ORs and general wards [12]; further, most studies on practical education needs have been conducted only on nursing students or new nurses [13,14,15,16]. Studies of clinical nurses have shown that individual nursing performance competencies and practical education needs can differ depending on the track of nurses’ clinical careers (such as years of experience and type of position) [17,18,19,20]. In particular, Seo et al. [21] reported that nursing competencies were significantly higher when they had more than 11 years of clinical experience and worked in intensive care units. In addition, education needs among nurses and related professions can differ depending on the clinical environment in which nurses operate and their level of nursing competency [18].

OR nurses are considered to have different practical education needs according to their clinical careers because they need to develop highly complex nursing performance competencies, different from those of other nurses in general wards [22,23]. Nevertheless, to the best of our knowledge, few studies have investigated nursing performance competence and practical education needs according to clinical career in only OR nurses. Considering this, it may be necessary to analyze whether the nursing performance competencies and practical education needs of OR nurses differ according to their clinical careers. Therefore, the aims of this study were to: (i) identify the level of nursing performance competencies and practical education needs; (ii) identify the nursing performance competencies and practical education needs according to demographic characteristics; (iii) identify the nursing performance competencies and practical education needs according to clinical career; and (iv) examine the correlation between nursing performance competency and practical education needs in terms of clinical career among OR nurses.

## 2. Method

### 2.1. Study Design, Sample, and Data Collection

This study is a descriptive cross-sectional study that aimed to compare nursing performance competencies and practical education needs among OR nurses according to clinical careers. The participants were nurses who were working in ORs as scrub or circulating nurses after having completed training under a preceptor. The sample pool was recruited using convenience sampling from four university hospitals located in Seoul, Korea, each of which had over 300 beds. A power analysis conducted using the G*Power 3.1.4 program (Heinrich-Heine-University, Düsseldorf, Germany) indicated that a sample of at least 180 participants would be necessary to reach a significance level of 0.05, 80% power, and a medium effect size of 0.25 for an analysis of variance examining the differences among four groups [24]. Considering this and allowing for potential incomplete responses, we distributed 200 questionnaires across the four university hospitals between 28 May 2018 and 16 June 2018, 192 of which were completed and returned (return rate: 96%). After excluding incomplete questionnaires, data for 182 participants were used for the final analyses. Therefore, the sample examined was an appropriate size.

The researcher explained the purpose of the study to the directors of the respective nursing departments. The researcher then distributed the questionnaires (which provided information regarding the study on the front page) to potential respondents, and after reading it OR nurses who consented to participate signed their names on the front page, confirming their understanding of the study’s purpose. After the participants returned their consent forms, data collection proceeded using self-report questionnaires.

### 2.2. Measurements

#### 2.2.1. Nursing Performance Competencies

Nursing performance competency was measured using a tool modified for OR nurses by Choi and Eun [25], which was an amended version of a tool used in previous studies [26,27]. This tool contains 23 items and measures 12 domains: “professional development” (scrub nurse’s role, circulating nurse’s role, and attendance at education programs or meetings), “inclination toward ethical value” (protection of patient’s rights and understanding the patient’s complaints), “cooperation” (establishing relationships with nurses and medical personnel), “developing others” (education and counseling, and the preceptor’s role), “self-control” (resolution of conflict with medical personnel), “self-confidence” (self-confidence of work performance and competency of new work management), “influence” (influence on other medical personnel), “resource management” (managing equipment and materials, including aseptic materials), “clinical judgment and measurement” (observing the operation procedure), “safety measurement” (managing the safety environment, preventing medical error during operation, and infection control), “processing ability in the operating room” (managing the operating room and taking over duties), and “flexibility” (flexibility and pursuit of information). Responses were provided using a five-point Likert scale, and mean scores were calculated for each dimension. Higher scores indicate higher nursing performance competencies. In the study by Choi and Eun [25], Cronbach’s α was 0.96. For this study, Cronbach’s α was also 0.96.

#### 2.2.2. Practical Education Needs

Practical education needs were measured using a tool modified by Kim [28], which is an amended version of a tool used in previous studies [29,30]. This tool contains 41 items and measures six domains: “preoperative patient identification” (six items), “counts” (six items), “prevention of transmissible infections” (14 items), “injury prevention” (six items), “management of surgical equipment” (five items), and “specimen management” (four items). Responses were provided using a five-point Likert scale, and mean scores were calculated for each dimension. Higher scores indicate higher practical training needs. In the study by Jang et al. [29], Cronbach’s α was 0.97; in the study by Kim [30], it was 0.96. For this study, Cronbach’s α was also 0.97.

Demographic characteristics of study participants included age (<30 years, 30–39 years, and ≥40 years), gender (female and male), educational status (diploma, bachelor’s, and beyond master’s), job position (staff, charge, or head nurse), and experience as preceptor (yes or no). Accordingly, using the clinical career structure developed by Jang [26], which models the progression from novice to expert across five steps (novice, advanced beginner, competence, proficiency, expertise) and is based on the “Dreyfus model of skill acquisition” by Benner [31], this study sought to analyze four groups of OR nurses, distinguished based on their career length: ≥1 year career, 1–3 years, 3–7 years, and >7 years.

### 2.3. Data Analysis

Data were analyzed using IBM SPSS Statistics Version 23.0 (SPSS, Inc., Chicago, IL, USA). Before analysis, the data were examined for outlier and missing responses. We then calculated means and standard deviations of participants’ scores regarding nursing performance competencies and practical educational needs. Next, we conducted *t*-tests and one-way analyses of variance on nursing performance competencies and practical education needs in terms of demographic characteristics and clinical career, and we performed post hoc analyses using Scheffe’s test. Finally, correlations among scores for nursing performance competencies and practical education needs in terms of clinical career were examined using Pearson’s correlation coefficient. *p* < 0.05 was considered to indicate significance.

### 2.4. Ethical Consideration

This study was approved by the institutional review board (IRB No. UC18QEDI0046) of the researcher’s hospital.

## 3. Results

### 3.1. Levels of Nursing Performance Competencies and Practical Education Needs

Table 1 presents the respondents’ levels for each domain of nursing performance competencies and practical education needs. The mean score for nursing performance competencies was 3.50. Here, *safety measurement* returned the highest score at 3.78, followed by cooperation (3.76), inclination toward ethical value (3.61), and professional development (3.56). Influence returned the lowest score at 3.19, followed by developing others (3.22) and self-confidence (3.24). Meanwhile, the mean score for practical education needs was 2.50. Here, management of surgical equipment returned the highest score at 2.98, followed by injury prevention (2.55) and prevention of transmissible infection (2.51). Preoperative patient identification returned the lowest score at 2.26, followed by counts (2.31) and specimen management (2.42).

### 3.2. Nursing Performance Competencies and Practical Education Needs by Demographic Characteristics and Clinical Career

Of the 182 participants included in the analyses, 34.6% were 30–39 years old, 85.2% were female, 65.3% had bachelor’s degrees, 67.1% were staff nurses, 38.4% had experience as a preceptor, and 44.5% had ≥7 years of experience in a clinical career (Table 2). Table 2 also presents the differences in nursing performance competencies and practical education needs according to demographic characteristics and clinical career. For nursing performance competencies, there were significant differences with regard to age group, educational status, job position, experience of preceptorship, and clinical career. For practical education needs, there were significant differences with regard to age group, gender, educational status, job position, experience of preceptorship, and clinical career.

### 3.3. Domains of Nursing Performance Competencies and Practical Education Need by Clinical Career

Table 3 presents the differences in each domain of nursing performance competencies and practical education needs by clinical career. Regarding nursing performance competencies, clinical career was found to effect significant differences in all domains, specifically, professional development, inclination toward ethical value, cooperation, developing others, self-control, self-confidence, influence, resource management, clinical judgment and measurement, safety measurement, processing ability in the operating room, and flexibility. Clinical career also effected significant differences in all domains of practical education needs: preoperative patient identification, counts, prevention of transmissible infections, injury prevention, management of surgical equipment, and specimen management.

### 3.4. Correlation between Nursing Performance Competencies and Practical Education Needs by Clinical Career

Table 4 presents the correlation between nursing performance competencies and practical education needs in terms of clinical career. This shows that the participants’ levels of nursing performance competencies were negatively correlated with their practical education needs. In particular, nursing performance competencies were negatively correlated with the ≤1 and >7 years groups.

## 4. Discussion

We identified that OR nurses’ nursing performance competencies and practical education needs differed significantly across clinical career groups. First, for nursing performance competencies according to clinical career among OR nurses, when compared to the other groups, the >7 years group showed significant differences in all categories and also scored the highest in all categories. The 1–3 and the 3–7 years career groups showed no significant differences when compared to the ≤1 year group in the categories of cooperation, inclination toward ethical value, processing ability in the operating room, flexibility, self-control, self-confidence, developing others, and influence. This result can be explained by the result of a previous study [32] that evaluated nursing performance competencies in terms of a nurse career ladder system and found that nurses in the higher rung group of the career ladder system had higher nursing performance competencies than did both those with higher and those with lower clinical career lengths. In other words, OR nurses with 1–7 year careers, who are no longer new nurses and who have begun to become accustomed to their jobs, can have differing performance competencies depending on their learning environments and personal development efforts even though they have equal career lengths. Therefore, in order to improve nursing performance competencies, it is necessary to take into account not only clinical career but environmental factors such as available educational environment support and career systems.

Next, the practical education needs of OR nurses differed significantly depending on their clinical career. In the results of our Scheffe’s post-hoc comparison, the ≤1 year group had the highest practical education needs, and the >7 years group had the lowest; there was no difference between the 1–3 and the 3–7 years career groups. This result is similar to our finding that nursing performance competencies differ depending on clinical career. Concurrently, all of the groups had high education needs regarding the management of surgical equipment. With the continuing development of medical technologies, advanced equipment that requires professional operation is continually being introduced and, as a result, the performance competencies of the OR nurses who manage the equipment require continuous improvement [1]. We suggest that OR nurses should continuously engage in up-to-date practical education programs on advanced equipment regardless of their clinical career.

Furthermore, OR nurses’ nursing performance competencies were negatively correlated with practical education needs in terms of clinical career. There was a negative correlation only between the ≤1 year group and the >7 years group. This may be because new nurses with less than one year of experience have differing practical education needs (such as becoming accustomed to the special conditions and jobs of the OR) and because members of the >7 years group are beginning to assume new jobs and responsibilities in the unit, such as becoming middle managers of senior nurses in ORs. In other words, the >7 years group requires new education in order to improve their competencies as middle managers; if they have low performance competency, they have higher practical education needs. In the continuous education system for middle managers that is employed in Korean hospitals, all nurses (regardless of unit) are expected to participate in a continuing nursing education program (valued at eight credits); however, whether it is an OR-related education program is not considered. Based on this study’s finding that OR nurses have differing nursing performance competencies and practical education needs according to their clinical career, it is clear that nursing managers must provide a variety of practical education programs that meet stepwise education needs, accommodating differences in clinical careers among OR nurses.

We also found that the higher their age, education, and position, the higher the levels of nursing performance competencies; in particular, nurses with experience as preceptors scored higher points in this study. This result accords with findings by Kang et al. [12], who reported that nurses in each unit perform more important jobs as they get older and their clinical career lengthens and that more participation in education, training, and workshops leads to an improvement in nursing performance competencies. In addition, we analyzed the influence that our respondents’ demographic characteristics had on their practical education needs. Consequently, we found that the lower the nurses’ age and education, the higher they scored in regard to practical education needs; head nurses’ education needs were higher than those of staff nurses, and nurses without preceptor course experience had the highest needs. Those aged 30 years and younger had particularly high practical education needs; this may be because of their need to adapt to the use of various surgical methods and equipment. The reason for higher needs among head nurses may be that they have higher perceived professionalism and require practical education regarding safety and management in ORs.

## 5. Limitations

There are several limitations to this study. It was conducted using convenience sampling in only four university hospitals with over 300 beds each. Therefore, caution should be exercised regarding the generalizability of the study results. Since we lacked a validated tool to measure the nursing performance competencies and practical education needs for OR nurses, we suggest further research on the development and validity of such a questionnaire. The study focused on the influence of differences among OR nurses’ clinical careers on nursing performance competencies and practical education needs. It is necessary to build on this by surveying nurses in other units regarding clinical-career-based influence on nursing performance competencies and practical education needs and by conducting a comparative analysis. Moreover, we suggest further studies to identify the direct and indirect effects through the mediator of the individual clinical career.

## 6. Conclusions

For OR nurses to accrue high levels of nursing performance competencies, it is important to provide an effective, evidence-based clinical nursing education program that reflects the practical education needs such nurses have at certain stages of their clinical careers. Our examination showed that for our group of new nurses, which comprised individuals with less than one year’s experience, and our middle-manager group, which comprised nurses with over seven years’ experience, the lower the level of nursing performance competencies, the higher the practical education needs. This result underlines the necessity of supporting sustainable education in hospitals and academic associations in order to achieve optimized and continuous education suited to the varying stages of professionals’ clinical careers.

## Figures and Tables

**Table 1:** Levels of nursing performance competencies and practical education needs (*n* = 182).

Variable	Min	Max	Mean ± SD
Nursing performance competencies	1.48	5.00	3.50 ± 0.67
Professional development	1.00	5.00	3.56 ± 0.77
Inclination toward ethical value	1.00	5.00	3.61 ± 0.85
Cooperation	2.00	5.00	3.76 ± 0.73
Developing others	1.00	5.00	3.22 ± 0.90
Self-control	1.00	5.00	3.42 ± 0.79
Self-confidence	1.50	5.00	3.24 ± 0.77
Influence	1.00	5.00	3.19 ± 0.79
Resource management	1.50	5.00	3.45 ± 0.78
Clinical judgment and measurement	1.00	5.00	3.36 ± 0.87
Safety measurement	2.00	5.00	3.78 ± 0.77
Processing ability in the operating room	1.50	5.00	3.50 ± 0.87
Flexibility	1.50	5.00	3.50 ± 0.75
Practical education needs	1.00	5.00	2.50 ± 1.25
Preoperative patient identification	1.00	5.00	2.26 ± 1.36
Counts	1.00	5.00	2.31 ± 1.44
Prevention of transmissible infections	1.00	5.00	2.51 ± 1.27
Injury prevention	1.00	5.00	2.55 ± 1.27
Management of surgical equipment	1.00	5.00	2.98 ± 1.30
Specimen management	1.00	5.00	2.42 ± 1.39

**Table 2:** Nursing performance competencies and practical education needs by demographic characteristics (*n* = 182).

Variable	Classification	*n* (%)	Nursing Performance Competencies			Practical Education Needs		
			Mean ± SD	t of F	*p* (Scheffe)	Mean ± SD	t of F	*p* (Scheffe)
Age	<30 ^a^	93 (51.1)	3.12 ± 0.57	61.91	<0.001	3.08 ± 1.14	26.30	<0.001
	30–39 ^b^	63 (34.6)	3.74 ± 0.50		(a < b < c)	1.95 ± 1.11		(a > b, c)
	≥40 ^c^	26 (14.3)	4.29 ± 0.32			1.75 ± 0.99		
Gender	Female ^a^	155 (85.2)	3.54 ± 0.61	1.46	0.155	2.41 ± 1.20	2.12	0.041
	Male ^b^	27 (14.8)	3.27 ± 0.92			3.03 ± 1.44		(a < b)
Education	Diploma ^a^	27 (14.9)	3.32 ± 0.78	24.79	<0.001	2.91 ± 1.51	8.43	<0.001
Status	Bachelor’s ^b^	119 (65.3)	3.35 ± 0.58		(a, b < c)	2.62 ± 1.18		(a, b > c)
	Master’s or above ^c^	36 (19.8)	4.13 ± 0.47			1.79 ± 1.01		
Job	Staff nurse ^a^	122 (67.1)	3.26 ± 0.62	37.17	<0.001	2.81 ± 0.12	15.86	<0.001
position	Charge nurse ^b^	57 (31.3)	3.97 ± 0.44		(a < b < c)	1.79 ± 1.03		(b < c)
	Head nurse ^c^	3 (1.6)	4.68 ± 0.18			3.42 ± 2.11		
Experience as preceptor	Yes ^a^	70 (38.4)	3.95 ± 0.51	8.44	<0.001	2.06 ± 1.22	3.88	<0.001
	No ^b^	112 (61.6)	3.22 ± 0.60		(a > b)	2.77 ± 1.20		(a < b)
Clinical career	≤1 year ^a^	25 (13.7)	2.77 ± 0.62	50.70	<0.001	3.60 ± 1.06	20.67	<0.001
	>1 to ≤3 years ^b^	34 (18.7)	3.06 ± 0.55		(a, b < c < d)	3.10 ± 1.23		(a > b, c > d)
	>3 to ≤7 years ^c^	42 (23.1)	3.40 ± 0.36			2.69 ± 1.26		
	>7 years ^d^	81 (44.5)	3.96 ± 0.50			1.81 ± 1.10		

SD: Standard deviation. a, b, c, d, F: one-way analysis of variance and Scheffe multiple comparison analysis tests.

**Table 3:** Domains of nursing performance competencies and practical education needs by clinical career (*n* = 182).

Domains	≤1 year ^a^	>1 to ≤ 3 years ^b^	> 3 to ≤ 7 years ^c^	> 7 years ^d^	F	*p*	(Scheffe)
	Mean ± SD	Mean ± SD	Mean ± SD	Mean ± SD			
**Nursing Performance Competencies**							
Professional development	2.63 ± 0.85	3.17 ± 0.65	3.45 ± 0.45	4.07 ± 0.50	48.68	<0.001	(a < b, c < d)
Inclination toward ethical value	3.24 ± 1.06	3.21 ± 0.75	3.36 ± 0.65	4.02 ± 0.72	14.30	<0.001	(a, b, c < d)
Cooperation	3.26 ± 0.75	3.50 ± 0.64	3.61 ± 0.62	4.09 ± 0.67	13.90	<0.001	(a, b, c < d)
Developing others	2.44 ± 0.71	2.51 ± 0.94	3.18 ± 0.57	3.78 ± 0.66	37.92	<0.001	(a, b < c < d)
Self-control	3.16 ± 0.85	3.06 ± 0.78	3.29 ± 0.71	3.72 ± 0.71	8.44	<0.001	(a, b < d)
Self-confidence	2.58 ± 0.69	2.90 ± 0.70	3.11 ± 0.50	3.65 ± 0.70	22.81	<0.001	(a < c < d)
Influence	2.60 ± 0.76	2.97 ± 0.90	3.05 ± 0.44	3.54 ± 0.74	13.59	<0.001	(a, b, c < d)
Resource management	2.54 ± 0.61	3.00 ± 0.63	3.39 ± 0.49	3.94 ± 0.62	44.95	<0.001	(a < b < c < d)
Clinical judgment and measurement	2.40 ± 0.82	2.91 ± 0.71	3.36 ± 0.73	3.85 ± 0.65	33.13	<0.001	(a < b, c < d)
Safety measurement	2.85 ± 0.59	3.32 ± 0.67	3.76 ± 0.55	4.27 ± 0.56	47.44	<0.001	(a < b < c < d)
Processing ability in the operating room	2.58 ± 0.61	2.84 ± 0.73	3.44 ± 0.55	4.09 ± 0.66	51.57	<0.001	(a, b < c < d)
Flexibility	2.92 ± 0.72	3.06 ± 0.65	3.39 ± 0.55	3.93 ± 0.63	25.40	<0.001	(a < c < d)
Practical education needs							
Preoperative patient identification	3.41 ± 1.16	3.00 ± 1.28	2.32 ± 1.32	1.58 ± 1.06	21.55	<0.001	(a > d)
Counts	3.45 ± 1.13	3.01 ± 1.44	2.42 ± 1.39	1.60 ± 1.14	18.83	<0.001	(a > b > d)
Prevention of transmissible infections	3.49 ± 0.97	3.08 ± 1.10	2.81 ± 1.26	1.80 ± 1.03	22.18	<0.001	(a > b, c, d)
Injury prevention	3.69 ± 0.97	3.07 ± 1.07	2.79 ± 1.23	1.86 ± 1.05	23.53	<0.001	(a > b > d)
Management of surgical equipment	4.06 ± 0.92	3.49 ± 1.09	3.29 ± 1.09	2.28 ± 1.20	21.60	<0.001	(a > d)
Specimen management	3.53 ± 1.24	2.99 ± 1.42	2.55 ± 1.30	1.78 ± 1.13	16.38	<0.001	(a > b > d)

SD: Standard deviation. a, b, c, d, F: one-way analysis of variance and Scheffe multiple comparison analysis tests.

**Table 4:** Correlation between nursing performance competencies and practical education needs in terms of clinical career (*n* = 182).

Variable	Practical Education Needs				
	Overall	≤1 year	>1 to ≤3 years	>3 to ≤7 years	>7 years
	r (*p*)	r (*p*)	r (*p*)	r (*p*)	r (*p*)
Nursing performance competencies	−0.532 (<0.001)	−0.404 (0.045)	−0.106 (0.549)	−0.180 (0.255)	−0.364 (0.001)
